# Genetic Evidence of Functional Ficolin-2 Haplotype as Susceptibility Factor in Cutaneous Leishmaniasis

**DOI:** 10.1371/journal.pone.0034113

**Published:** 2012-03-23

**Authors:** Amal Assaf, Tong Van Hoang, Imad Faik, Toni Aebischer, Peter G. Kremsner, Jürgen F. J. Kun, T. P. Velavan

**Affiliations:** 1 Institute of Tropical Medicine, University of Tübingen, Wilhelmstr. 27, Tübingen, Germany; 2 Department of Laboratory Medicine, Faculty of Medicine, University of Damascus, Damascus, Syria; 3 Robert Koch Institute; Nordufer 20, 13353 Berlin, Germany; University of Lausanne, Switzerland

## Abstract

**Background:**

Ficolin-2 coded by *FCN2* gene is a soluble serum protein that plays an important role in innate immunity. In this study, we analyzed five functional polymorphisms of the *FCN2* gene for their possible association with cutaneous leishmaniasis.

**Methods:**

Initially we screened 40 Syrian Arabs for the entire *FCN2* gene. We investigated the contribution of *FCN2* functional variants in 226 patients with cutaneous leishmaniasis and 286 healthy controls from Syria. Polymorphisms in the promoter regions (*−986G/A, −602G/A, −4A/G*) of the *FCN2* gene were assessed by TaqMan real time PCR, whereas polymorphisms in exon8 (*+6359C/T and +6424G/T*) were assessed by DNA sequencing. We also measured serum ficolin-2 levels in 70 control Syrian Arabs and correlated the serum concentrations to *FCN2* genotypes and haplotypes respectively.

**Results:**

Nine new *FCN2* variants including two with non synonymous substitutions in exon6 and exon8 were observed. The homozygous genotypes *+6424T/T* were distributed more in controls and none in patients (*P* = 0.04). The *AGACG* haplotype were observed more in patients than in controls (OR = 2.0, 95%CI 1.2–3.4, *P* = 0.006). The serum ficolin-2 levels were significantly distributed among the reconstructed ficolin-2 haplotypes (P<0.008) and the haplotype *AGACG* was observed with higher ficolin-2 levels in 70 control individuals.

**Conclusion:**

Our results demonstrate a significant association of *FCN2 AGACG* haplotype with cutaneous leishmaniasis in a Syrian Arab population. These first results provide a basis for a future study that could confirm or disprove possible relationships between *FCN2* gene polymorphisms with cutaneous leishmaniasis.

## Introduction

Leishmaniasis is caused by protozoan parasite of the genus Leishmania and poses a serious health threat in many tropical and subtropical countries with estimated 350 million people at risk and 12 million people affected. Despite considerable efforts to control the disease the incidence worldwide is still on the rise. Three distinct clinical forms, cutaneous (CL), visceral (VL) and mucocutaneous leishmaniasis (MCL) are classically caused by a spectrum of different *Leishmania* species [1]. Although there is a clear correlation between the causative species and the clinical presentation, many variations are observed depending on the immunocompetence of the host [2]. Resistance against leishmaniasis depends on a Th1-type response resulting in the production of interferon gamma whereas Th2-type response is related to susceptibility [1], [3]. Apart from the characteristics of the species and strains, the hosts innate immunity influences the severity of the disease to a great extent [4], [5]. Genome-wide studies have been performed to define quantitative trait loci (QTL) in mice and in humans with CL and VL caused by *L. major* [6]. Some of the identified QTL seems to be rather species-specific while a few play a role in other parasitic diseases [7].

In this study, we aim to investigate the contribution of ficolin-2 (*FCN2*) gene polymorphisms to the susceptibility for leishmaniasis caused by *L. tropica* in a Syrian cohort. Ficolin-2 is an important factor of the innate immune response and is located on chromosome 9q34 and contains eight exons [8]–[10]. Ficolin-2 binds to different pathogen–associated molecular patterns (PAMPs), such as carbohydrates, lipoteichoic acid and acetylated groups leading to the pathogen phagocytosis and activation of complement system through the lectin pathway [11]. The repertoire of microorganisms recognized by ficolin-2 may be as widespread as for the mannan–binding lectin (MBL). Recent studies have demonstrated MBL binds to *L. braziliensis* and the MBL binding is mediated by a specific carbohydrate on the parasite surface [12]. It has been recently shown that *FCN2* is highly polymorphic and that low levels of circulating ficolin-2 are clearly associated with polymorphisms in the promoter (*−986G/A, −602G/A, −4A/G*) and in exon 8 (*+6359C/T* and *+6424G/T*) of the gene [10], [13]. Although so far no total deficiency of ficolin-2 has been reported, low circulating levels of the protein have been shown to be associated with recurrent respiratory infections in children [14], to contribute to the severity of renal disease in IgA nephropathy [15] and in autoimmune diseases [16]. Also recent studies have investigated the possible associations between *FCN2* gene polymorphisms to ficolin-2 levels in rheumatic fever, rheumatic heart disease, leprosy, hepatitis B virus and malaria [16]–[19]. In the current study, we aim to investigate the role of five functional *FCN2* gene polymorphisms (*−986G/A, −602G/A, −4A/G, +6359C/T* and *+6424G/T*) for a possible association to the leishmaniasis outcome in a Syrian cohort. Further in the case control analysis, we aim to compare and relate any possible outcome caused either by the genotypes or by the haplotypes that were established to influence serum ficolin levels.

## Materials and Methods

### Ethics

Informed written consent was given by all participants. The study was approved by the local ethics committee of the University of Damascus and also approved by the Ministry of Health, Syria.

### Patients and sampling

A total of 226 patients from Syria presenting different clinical outcomes of cutaneous leishmaniasis characterised by a lesion (“Aleppo boil”; (87 (37%) females and 148 (63%) males, mean age 26±15 S.D years) were included in the study. They were consecutive outpatients from different dermatologic clinics in Syria. All patients were diagnosed with cutaneous leishmaniasis according to clinical features and Giemsa staining of dermal scrapings from affected lesions. Also 286 healthy unrelated symptom-free subjects were assessed as controls (87 (38%) females and 145 (62%) males, mean age 26±13 S. D years). Both patients and controls belonged to the same social status, geographical area and Arabic ethnic background. The participants of the study were recruited between March 2008 and March 2009 and in December 2011. All patients and control subjects signed an informed written consent.

### Genomic DNA isolation and *FCN2* genotyping

Three ml of venous whole blood was drawn from the study subjects. DNA extraction was performed using QIAamp™ DNA extraction kits following the manufacturer's instructions (QIAGEN GmbH, Hilden, Germany). The entire coding regions of the gene were amplified in 40 control individuals and were analyzed by direct DNA sequencing. DNA was amplified using a primer pair spanning one or two exons (Table 1). In brief: 100 ng of genomic DNA was amplified in a 25 µl volume of reaction mixture containing 1× reaction buffer (20 mM Tris pH 8.8, 10 mM KCl, 1.5 mM MgCl_2_ and 0. 1% Triton X-100), 1× Q-solution (QIAGEN), 0.2 mM dNTPs, 0.5 mM MgCl_2_, 0.5 µM of each primer and 1.0 U Taq polymerase (QIAGEN). The cycling conditions used for amplification were 95°C for 5 min; 35 cycles of 95°C for 40 s, 59°C for 60 s, 72°C for 90 s, and with a final extension of 72°C for 3 min. The amplified PCR fragments were stained with SyBrgreen I (Biozym Diagnostik GmbH, Wien, Austria) and visualized on a 1% agarose gel. The PCR products were purified by using the EZNA Cycle-Pure kit following manufacturer's instructions (PeqLab Biotechnologie GmbH, Erlangen, Germany). Purified PCR products were then sequenced with the BigDye® Terminator v1. 1 Cycle Sequencing Kit (Applied Biosystems, Foster City, CA, USA). Sequencing reactions were analysed on an automated sequencer (ABI Prism 3100 Genetic Analyzer, Applied Biosystems). The resulting DNA sequences were aligned using Bio-Edit software, and DNA polymorphisms were confirmed visually from sequence electropherograms. The identified novel polymorphisms were submitted to the SNPper database and appropriate SNP #rs IDs were received.

**Table 1:** Primer pairs utilized for characterization of the entire *FCN2* gene in 40 Syrian Arabs.

Reverse primer	Forward primer	
5′- gaa gcc acc aat cac gaa g- 3′	5′- att gaa gga aaa tcc gat ggg- 3′	Promoter+Exon 1
5′- gtt cct ctg cag cca ggt c- 3′	5′- aga tgg cag atg cct ttc ag- 3′	Exon 2+3
5′- agg ctc ttg tgt tcc agg c- 3′	5′- agg ccc aga aaa tgg tgt c- 3′	Exon 4+6
	5′-ata cag acg cct atg gcc c- 3′	Exon 5*
5′- tta caa acc gta ggg cca ag- 3′	5′- cca gct ccc atg tct aaa gg- 3′	Exon 7+8

* used only for sequencing.

The determination of polymorphism at *−986G>A, −602G>A* and *−4A>G* in the promoter regions were investigated by TaqMan-Real-Time PCR (TaqMan MGE assay, Applied Biosystems, Foster City, CA, USA), whereas for the positions *+6359C>T* and *+6424G>T* DNA sequencing method was employed. The TaqMan-Real-Time PCR technique is based on amplification of the region flanking the SNP in the presence of two allele-specific fluorescent probes, of which the 5′end is labelled with a reporter dye (either FAM or YAK) and the 3′ end is labelled with a quencher dye (Table 2). Both alleles can be detected in a single tube. The probes do not fluoridate in solution because of the quencher activity at the 3′end. During the PCR, the exact matching hybridization probe remains bound and is degraded. Then the fluorophore is released and detected. A mismatch probe abolishes the hybridization of the probe and no degradation and subsequently no fluorophore can be detected.

**Table 2:** Primers and probes employed for genotyping three promoter SNPs in the *FCN2* gene.

Position	Primer/Probe	Sequence
−986G>A	Primers	5′-tgatcttgccaaggaagaaggc-3′
		5′- ccactaccaccaccgca -3′
	Probes	5′-YAK-acctgccgccatcgg- BBQ3
		5′ -FAM-acctgctgccatcggga- BBQ3′
−602G>A	Primers	5′-tccccactcttctctcctttcc-3′
		5′-cctggggcagtatgtagagca-3′
	Probes	5′-YAK-tcctgttcgtgtgcccc-BBQ3′
		5′-FAM-tcctgttcatgtgcccctg-BBQ3′
−4A>G	Primers	5′- aagatgagaaattggagtctgaggga- 3′
		5′-gaaagagagcagcagggtgg-3′
	Probes	5′-YAK- ctccatctcctctggtctttgctt - BBQ3′
		5′-FAM- agctccatctcttctggtctttgc - BBQ3′

YAK and FAM are the reporter fluorophores Yakima Yellow and Fluorescein, respectively.

Each probe carries at the 3′-end the dark BBQ (BlackBerry Quencher).

For each allele-specific reaction, 12.5 µl of TaqMan Universal Master Mix, 5 mM of forward and reverse primer, 1 µl of probe labelled with 10 mM FAM and 1 µl of probe labelled with 10 mM YAK and 20 ng of DNA diluted in 6.5 µl of H_2_O were dispensed under the following conditions in a 0.1 ml tube for each reaction (Corbett Research LTF-Labortechnik GmbH, Wasserburg, Germany): polymerase activation for 15 minute at 95°C 40 two-step cycles consisting of: 60 s of denaturation at 94°C followed by 1 minute of annealing and elongation at 60°C. Results were analyzed on Rotor-Gene 3000 using Rotor-Gene software v 6. 1 (Corbett Life Science, Australia). Genotype results were manually assigned.

### Ficolin-2 ELISA

The ficolin-2 concentrations were measured in sera in 70 Syrian Arab controls using the human ficolin-2 ELISA kit (Hycult Biotech Cat.#: HK336, Uden, The Netherlands) according to the manufacturer's instructions. The minimum ficolin-2 concentrations that can be measured by this kit are 16 ng/mL.

### Statistical analysis

Data had been analysed by STATA (Stata Corporation, Texas, USA) and the level of significance was set to P<0.05. Normal Chi square, Kruskal-Wallis test and two tailed Fisher's exact tests were executed to determine the differences in genotype, haplotype and ficolin-2 level distributions. Genotype or haplotype frequencies were determined by simple gene counting and by using the expectation-maximum (EM) algorithm. The significance of deviations from Hardy-Weinberg equilibrium and exact tests for population differentiation was tested using the random-permutation procedure and by default Markov chain parameters as implemented in the Arlequin v. 3.5.1.2 software (http://cmpg.unibe.ch/software/arlequin3/). Linkage disequilibrium (LD) analysis was performed using Haploview v. 3.2 program.

## Results

**Figure 1:**
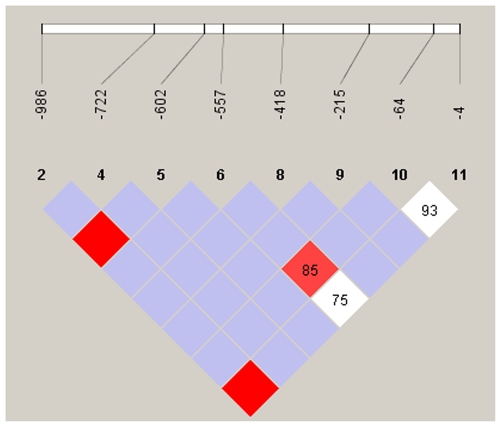
Haploview plot illustrating the linkage disequilibrium of the *FCN2* promoter region from 40 healthy Syrian individuals. At the top the SNPs are shown according to their succession from the start of translation of the *FCN2* gene. *Empty squares* indicate a high degree of LD (LD coefficient D′ = 1) between pairs of markers. Numbers indicate the D′ value expressed as a percentile. *Red squares* indicate pairs in strong LD with LOD scores for LD ≥2; purple squares, D′ = 1 with LOD = 2; *white squares*, D′ <1.0 and LOD ≤2.

**Figure 2:**
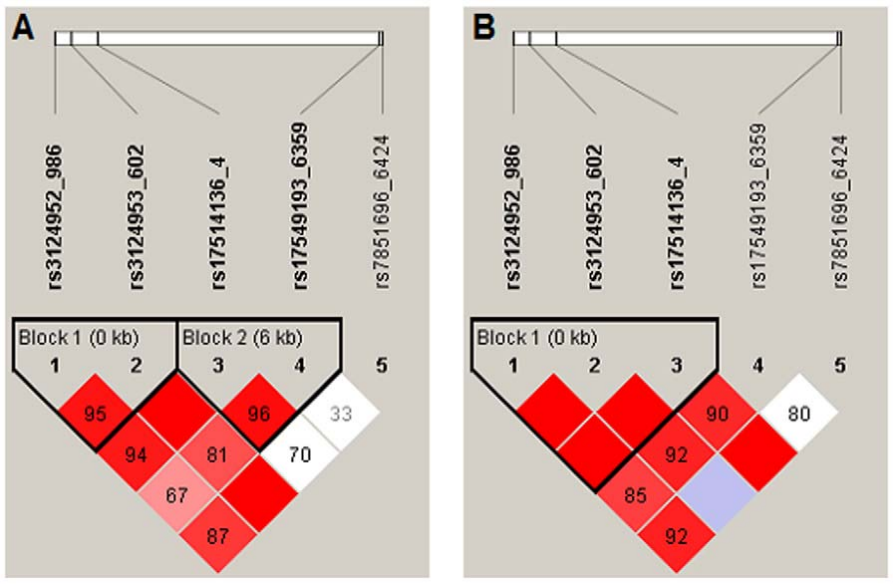
Haploview plot illustrating the linkage disequilibrium of the *FCN2* functional variants in controls (2A) and patients (2B). At the top the SNPs are shown according to their succession from the start of translation of the *FCN2* gene. *Empty squares* indicate a high degree of LD (LD coefficient D′ = 1) between pairs of markers. Numbers indicate the D′ value expressed as a percentile. *Red squares* indicate pairs in strong LD with LOD scores for LD ≥70; purple squares, D′ = 1 with LOD >1; *white squares*, D′ <1.0 and LOD ≤2.

**Figure 3:**
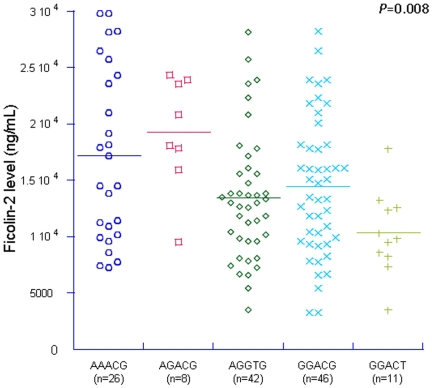
Distribution of serum ficolin levels to five major haplotypes (−986/−602/−4/+6359/+6424) in 70 control individuals.

Initial screening of 40 control subjects revealed nine novel SNPs: two in the promoter (*−722C>T*, *−418G>A*), one in intron2 (*+1898G>C*), four in intron5 (*+4577C>G, +4647C>T, +4704G>C, +4806G>A*), one in exon6 (*+4986C>T*) causing amino acid substitution from Arg to Trp and one in exon8 (+*6584G>A*) causing a change at position 311 from Arg to Gln (Table 3). The limited group size does not allow any analysis for low frequency SNPs. For the patient-control cohort, we analysed three functional SNPs in the promoter region (*−986G>A, −602G>A and −4A>G*) and two in exon8 (*+6359C>T* and *+6424G>T*). The distribution of five investigated functional SNPs was homogeneous in both patients and controls. Both genotype and allele frequencies for all analysed SNP variants (*−986G>A, −602G>A, −4A>G, +6359C>T, +6424G>T*) in controls and patients were in Hardy-Weinberg equilibrium. When looked at the *+6424G>T* variant in exon8 we found a statistically significant difference in genotypes between patients and controls: in the control cohort we found five individuals among 286 homozygous for *+6424T/T* (resulting in Ser at position 258 in the protein and leading to a low ficolin-2 level) but none among individuals in the patient cohort (*P* = 0.04). The loci (*−986G>A* and *−4A>G,*) and (*−557A>G* and *−64A>C*) in the Syrian cohort were highly linked to each other (Figure 1). Linkage disequilibrium analysis revealed strong allelic combinations at positions (*−986G>A, and −602G>A) and (−4A>G,* and *+6359C>T)* in the controls (Figure 2A) and *−986G>A, −602G>A and −4A>G* in CL patients (Figure 2B). The *AGACG* (*−986G>A, −602G>A, −4A>G, +6359C>T, +6424G>T*) haplotype was associated with CL. The *AGACG* haplotype was observed more in patients than in the controls (9.3% vs. 4.9%, OR = 2.0, 95%CI 1.2–3.4, p = 0.006) (Table 4). No significant distribution was observed between patients and controls when the haplotypes associated with higher ficolin levels (*AAAG+AGGG+AGAG+GGGG*) were compared to haplotypes associated with lower ficolin levels (*GGAT*). Also when haplotypes associated with medium ficolin levels (*GGAG*) were included to haplotypes associated with higher ficolin levels (*AAAG+AGGG+AGAG+GGGG*); no significant association was observed between patients and controls (Data not shown). Two *FCN2* genotypes (*−602G>A* and *+6424G>T*) were correlated to serum ficolin-2 levels in a gene dose dependent manner i.e. homozygotes had either the highest or the lowest Ficolin-2 concentrations, whereas heterozygotes had intermediate concentrations (Data not shown). The serum ficolin 2 levels were significantly distributed among the reconstructed ficolin2 haplotypes (P<0.008) (Figure 3).

**Table 3:** Characterization of the entire *FCN2* gene in 40 Syrian Arabs.

Base position	SNP rs#.	Major allele	Minor allele	Major allele	Minor allele
−986	rs3124952	G	A	0. 50	0. 50
**−722**	rs76739162	C	T	0. 99	0. 01
−602	rs3124953	G	A	0. 71	0. 29
−557	rs3811140	A	G	0. 73	0. 27
**−418**	rs74693003	G	A	0. 99	0. 01
**−214**	rs12344051	G	A	0. 97	0. 03
−64	rs28969369	A	C	0. 86	0. 14
−4	rs17514136	A	G	0. 81	0. 19
1878	rs3124955	T	C	0. 62	0. 38
**1898**	rs75577478	G	C	0. 97	0. 03
2051	rs73565973	T	C	0. 90	0. 10
2088	rs73565979	C	T	0. 90	0. 10
2182	rs12344423	G	A	0. 51	0. 49
2417	rs7024491	A	G	0. 66	0. 34
2472	rs3128624	A	G	0. 63	0. 37
2488	rs4520243	T	C	0. 62	0. 38
2545	rs7037264	G	A	0. 63	0. 37
**4577**	rs76665625	C	G	0. 99	0. 01
**4647**	rs75123259	C	T	0. 99	0. 01
**4704**	rs56117058	G	C	0. 86	0. 14
**4806**	rs77254375	G	A	0. 99	0. 01
4888	rs56200327	C	T	0. 99	0. 01
6031	rs11103563	A	G	0. 97	0. 03
6183	rs62573178	G	A	0. 92	0. 08
6220	rs7872508	T	G	0. 97	0. 03
6359	rs17549193	C	T	0. 81	0. 19
6424	rs7851696	G	T	0. 97	0. 03
**6584**	rs76267164	G	A	0. 99	0. 01

**In bold** = new SNPs.

**Table 4:** *FCN2* haplotypes in Cutaneous Leishmaniasis patients and healthy controls.

*FCN2 haploytpes (−986/−602/−4/+6359/+6424)*	Patients n = 452 (%)	Healthy controls n = 572 (%)	OR (95% CI)	*P*
AAACG	79 (17.5)	118 (20.6)	NS	
GGACG	160 (35.4)	206 (36.0)	NS	
AGGTG	116 (25.7)	136 (23.8)	NS	
**AGACG**	**42 (9.3)**	**28 (4.9)**	**2.0 (1.2–3.4)**	**0.006**
GGACT	37 (8.2)	46 (8.0)	NS	
AGGCG	9 (2.0)	3 (0.5)	NS	
GGATG	6 (1.3)	15 (2.6)	NS	
GGATT	1 (0.2)	6 (1.0)	NS	
AGATG	0	3 (0.5)	NS	
AAATG	1 (0.2)	4 (0.7)	NS	
GGGTG	0	3 (0.5)	NS	
AGGTT	0	2 (0.3)	NS	
GAACG	0	2 (0.3)	NS	
AAACT	1 (0.2)	0	NS	

## Discussion

In Syria, 85% of cutaneous leishmaniasis is caused by *L. tropica*, the remaining 15% stem from *L. major*. Visceral leishmaniasis can be found in Syria but only in very low numbers and usually full blown disease does not develop [20]. In contrast, CL is found in larger numbers and is on the rise at times [21], [22]. For these two reasons we restricted our study to CL. Three main components of the host–parasite interaction in leishmaniasis determine the disease outcome: the host's genetic background, the immunological response and the parasite's intrinsic pathogenicity. Many authors have studied the influence of parasite strain and species on disease [23]. Fewer data are available on the influence of host factors. An association of functional polymorphisms in four cytokine genes with susceptibility to, and clinical outcome of cutaneous leishmaniasis has been shown, suggesting that functional genetic variants in the interleukin 4 (*IL4*) promoter could influence the risk of developing cutaneous leishmaniasis while the polymorphism in the first intron of the interferon (*IFN*)-gamma gene might influence the progression of disease towards chronic cutaneous leishmaniasis [24]. Genome-wide scan analyses have identified different chromosomal regions, which are involved in the development of different clinical forms for leishmaniasis. The findings indicate that multiple genes may control this immune response, probably with a strong ethnic component as an additive factor [25]–[28].

Considering the physiopathology of *Leishmania* infections, susceptibility to leishmaniasis seems to depend on early mechanisms that provide the interaction of *Leishmania* and phagocytes/macrophages [5]. Monocytes/macrophages provide the cellular habitat for the parasite and phagocytosis remains a vital step in the establishment of infection. Ficolin-2 and MBL are host opsonins that bind to surface of various pathogens and interact with the same molecules to be taken up by phagocytes [29], [30]. Followed by the entry of *Leishmania* into the host cell the cytokine response is modulated to favour *IL6* secretion which inhibits the antiparasitic properties of macrophages [31]. High level of MBL and associated genetic variants of *MBL2* haplotypes are therefore found more frequently in leishmaniasis patients than in healthy controls [32]. Hence MBL-deficient individuals are shown to be protected against entry of *Leishmania* to the host cells; this heterosis effect has also been observed for bacterial infections [33], [34]. However, we were not able to show binding of ficolin-2 to *Leishmania* surfaces as it was demonstrated for Trypanosoma with subsequent killing of the parasites [35]. The reasons for this may be of technical or methodological difficulties.

Since ficolins exert similar functions as MBL, it is conceivable that ficolin-2 may be of biological significance in the host interaction with *Leishmania* and as such has a role both in the initiation as well as in the clinical outcome of the disease. Thus ficolin-2 insufficiencies might play an advantageous role in the protection against *Leishmania* as do *MBL2* polymorphisms for bacterial infections [33], [34]. Since we do not have access to Syrian patient samples to determine the contribution of *FCN2* variants to serum ficolin 2 levels, we sampled additional seventy healthy control individuals and correlated their ficolin-2 serum levels with respective genotype and haplotype distribution. We observed no significant distribution between serum ficolin-2 levels and *FCN2* functional genotypes. Earlier studies have documented that ficolin-2 serum concentration was distributed in a gene dose-dependent manner, i.e. homozygotes had either the highest or the lowest ficolin-2 concentrations, whereas heterozygotes had intermediate concentrations [10]. In line with the documented studies, a similar trend was observed for the two *FCN2* functional genotypes (*−602G>A and +6424G>T*) to serum ficolin-2 levels. The lack of significant distribution may possibly due to lower sample size employed (n = 70). Nevertheless, a significant distribution among the reconstructed *FCN2* haplotypes was observed (*P*<0.008). Studies have documented a significant association of *FCN2* promoter genotypes (*−986G>A, −602G>A and −4A>G*) to serum ficolin levels in a Danish population and also on significant associations of the *FCN2* haplotypes (*AAAG, AGAG, AGGG, GGAG and GGAT*) with serum ficolin-2 levels in Gabonese children with mild malaria and the *AAAG* haplotype was associated with the highest ficolin levels [13], [36]. As observed from other studies, we do not observe a significant contribution of the *FCN2* genotypes; however, we observed that the *AGACG* haplotypes contributed to increased susceptibility to cutaneous Leishmaniasis. When looked at the *+6424G>T* variant in exon8, we found a statistically significant difference in genotypes between patients and controls. Also the *AGACG* haplotype contributed towards increased susceptibility to cutaneous leishmaniasis. These first results in the Arab population with CL provide a basis for a future study that could confirm or disprove these possible relationships. We also found a statistically significant difference in *+6424G>T* variant between patients and controls. Studies have demonstrated that the *+6424G>T* variant is associated with low ficolin 2 protein concentration and *+6359C>T* has lower GlcNAc binding capacity and is much more frequent than the *+6424G>T* variant [10]. We observed these *+6424G>T* variants more in patients that signifies an increased lectin (GlcNAc-binding) activity in patient population which effectively means the mechanism to thwart the CL infection is happening. No significant association was observed between haplotypes producing higher ficolin levels and that of haplotypes with lower ficolin levels. Of which the *AGAG* haplotype (higher ficolin-2 level producer) that was observed marginally significant in patients does not reflect actual higher ficolin levels. Also the *+6424G>T* variant (associated to lower ficolin levels) found significantly higher in patients does not imply a factual outcome on ficolin-2 levels. These associations have to be confirmed by measuring the serum ficolin levels in this cohort.

The frequency of the studied SNPs in the Arabs resembles largely the European distribution [37], except for *−557A>G* and *−64A>C*, that were 0.27 and 0.14 in frequency in the Syrian population compared to 0.12 and 0.02 in Danish population. The population differentiation tests computed for genotypes that were highly linked (*−557A>G* and *−64A>C*) in Syrian population were observed to be significantly different to that of Danish genotypes (P = 0.001) [13]. It seems therefore that selective forces have exerted positive selective pressure on *FCN2* haplotypes that would have conferred protection against some diseases. Although cutaneous leishmaniasis does not lead to mortality, an influence on the gene pool can easily be envisaged by the strong social stigmatization of leishmaniasis patients, which can lead to social exclusion including divorce [38], [39]. Also, human beings affected with severe skin disfigurations suffer a serious loss of quality of life that may lead to suicidal ideation and suicide itself [40]–[42]. Of course, it might be possible that other fatal diseases like tuberculosis exerts the selective force on the *FCN2* polymorphisms and *Leishmania* profits from this selective process. Studies on this subject are not yet available. In order to validate whether *FCN2* polymorphisms are as a result of selection or stochastic factors, it is essential to look at a larger population.

Overall, we present evidence that *FCN2* polymorphisms may be an additional factor contributing to the susceptibility to leishmaniasis. Although the role of ficolin-2 in the interaction with the *Leishmania* surface remains to be established, these results indicate a role for this molecule in leishmaniasis and represent another example how seemingly disadvantageous polymorphism can be beneficial in a population. The association of the specific haplotype is rather weak and the number of patients is low therefore additional studies with larger cohorts having more severe forms of leishmaniasis would be desirable.
